# Spotlight on Feline Oncology

**DOI:** 10.3390/vetsci10040246

**Published:** 2023-03-24

**Authors:** Louise van der Weyden

**Affiliations:** Sanger Institute, Wellcome Genome Campus, Hinxton, Cambridge CB10 1SA, UK; lvdw@sanger.ac.uk; Tel.: +44-1223-834-244

Cancer is a significant cause of morbidity and mortality in felines, with the majority of tumours (53–85% cases) being diagnosed as malignant [1] and often associated with a poor outcome. Sadly, however, there is a disproportionately lower amount of research into feline versus canine oncology, and in contrast to dogs, cats are not as commonly thought of as a model for human cancer. Shining a spotlight on our current understanding of feline oncology will hopefully serve as a springboard for further much-needed research into this field. This *Veterinary Sciences* Special Issue, entitled “*Spotlight on Feline Oncology*”, investigates this topic through twelve publications: six case reports, including both captive wild felids and domestic cats, three research papers and three reviews, all dedicated to broadening our understanding of cancer in cats, from detailed clinical presentations of a wide range of tumour types, to discussing the underlying causes and investigation of different treatment options.

There are two case reports on tumours in captive wild felids, specifically tigers. One details the pathological and immunohistochemical features of an ovarian leiomyoma that was an incidental finding in a white tiger (*Panthera tigris*) that had been treated with a gonadotropin-releasing hormone agonist to control reproduction, and suggests that further investigation of the role of contraceptives in the pathogenesis of cancer in non-domestic felids is warranted [2]. The other reports a sinonasal meningioma in a Siberian tiger (*Panthera tigris altaica*), providing details of the clinical presentation, gross pathology and histopathological findings observed in this case and highlighting the similarities to that seen in meningiomas of domestic cats; a non-infiltrative tumour with a clinically silent phenotype [3].

Four case reports involve tumours in domestic felids: two describing tumours with unusual presentations and two describing tumour responses to different treatment regimes. One is a report of a white-coated domestic short hair (DSH) cat with a primary fibrosarcoma on the convex surface of both auricles, in which detailed histopathological analysis revealed changes associated with chronic UV exposure, warranting further investigation into the correlation between the UV exposure and the occurrence of cutaneous non-epithelial (mesenchymal) tumours [4]. In another report, a DSH cat was presented for necropsy due to sudden death despite no prior clinical symptoms, and Toma and colleagues provide a detailed post-mortem evaluation of haemangiosarcoma of the pancreas (which is an unusual location for this tumour type) with abdominal metastasis and hemoperitoneum, as well as a comparative assessment of three anti-CD31 antibodies that can be used for immunohistochemical diagnosis of haemangiosarcoma [5]. The report of a Norwegian cat with vertebral osteosarcoma (OSA) that was treated with marginal surgical excision (debulking and a right hemilaminectomy) and chemotherapy (four cycles of doxorubicin followed by toceranib phosphate) is the first to document the complete resolution of vertebral OSA lesions using this protocol, and at 16 months post-treatment the cat showed no neurological abnormalities or signs of cancer recurrence [6]. A report of three DSH cats with B-cell nasal lymphoma with low mitotic index that were treated with chemotherapy (chlorambucil and prednisolone) revealed that two of the cases (with a mitotic index of 0–1 per ×400 field) achieved a prolonged disease-free interval, in contrast to the one with the highest mitotic index (mitotic index of 3–4 per ×400 field), suggesting that protocols using chlorambucil and prednisolone warrant further investigation as a first-line therapy for feline nasal lymphoma cases with a very low mitotic index [7].

The Special Issue also contains three reviews which cover a variety of topics, including aetiological factors for feline oral squamous cell carcinoma (FOSCC), infectious causes of neoplasia in felines and the genetics of feline cancers. FOSCC is the most common oral neoplasia in cats, and is a locally invasive tumour with a high mortality rate [8,9]. Sequeira and colleagues critically review the literature for potential aetiological factors of FOSCC, including viral infections; environmental factors, such as tobacco smoke exposure, diet, deworming methods and living environment conditions; as well as associated co-morbidities [10]. In recent years, there has been growing attention on the role that infectious agents play in tumour development and progression, and Rolph and Cavanaugh summarise the literature to date on the direct role that infectious organisms, such as viruses, bacteria and parasites, can play in the development of neoplasia, as well as the role they can play in predisposing the individual to neoplasia [11]. Similarly, there is a growing desire to characterise the genetics of feline tumours, and Ludwig and colleagues review the genetic investigations that have been carried out in both common and rare feline tumours, ranging from studies using cytogenetics to look for chromosomal changes, to studies using a single-gene approach by examining the mutation or expression status of a specific cancer gene, to more recent studies that are taking advantage of the advent of a high-quality *Felis catus* reference genome that is now available [12] and using this for next-generation sequencing-based analyses [1].

Finally, three research articles are presented, assessing patterns of lymphocytic infiltrates to distinguish between feline hepatic lymphoma and lymphocytic portal hepatitis, characterisation of the expression of angiogenic factors in cutaneous squamous cell carcinoma (cSCC) of cats and pathological findings and miRNA analyses in feline gastrointestinal masses (neoplasms and polyps). Lymphoma is the most common hepatic tumour in cats, and as with lymphomas at other sites histopathology can be challenging to distinguish between lymphocytic inflammation or a lymphoma [13]. To aid in this diagnostic challenge, Sebastian and colleagues performed a retrospective study on 44 feline liver biopsies and defined a specific set of visual patterns of lymphocytic infiltrates which are predictive of a lymphoma or inflammation [14]. cSCC is a common tumour in cats, and since angiogenesis is one of the key contributors to tumour growth and metastasis in humans, Gudenschwager-Basso and colleagues characterised the expression of key angiogenesis-promoting genes, VEGF-A, PLGF and their receptors (Flt-1, sFlt-1, KDR) in cSCC samples from cats to better understand the mechanisms of neovascularisation in feline cSCC [15]. Gastrointestinal masses in cats are of great clinical relevance and histological examination of the lesion is typically essential for diagnosis [16]; however, to date, there have been no large-scale studies investigating the pathology of feline gastro-intestinal masses. To this end, Kehl and colleagues performed a retrospective analysis of the pathology of 860 feline gastrointestinal masses, and found that the histopathology and immunohistochemistry revealed a range of different diagnoses, with the most common being lymphomas, carcinomas and spindle cell tumours (with different subtypes of each), which differed in their relative prevalence depending on tissue site [17]. In addition, they also analysed the expression of two microRNAs (miR-20b and miR-192), which are differentially expressed in canine intestinal T-cell lymphoma, to assess their diagnostic potential in feline intestinal cancer [17].

Taken together, this Special Issue offers a rich insight into our current understanding of the aetiology, molecular genetics, clinical presentation, histopathology and treatment options for cancer in felines.
